# A Subjective Assessment of Chemotherapy Drug-Induced Taste and Smell Alteration in Non-head and Neck Cancer Patients: A Questionnaire-Based Cross-Sectional Study

**DOI:** 10.7759/cureus.57787

**Published:** 2024-04-07

**Authors:** Nidhi Yadav, Swati Mittal, Prashanthi Reddy, Ajay Parihar, Saloni Agrawal, Rashi Mandlik, Arun D Sharma

**Affiliations:** 1 Oral Medicine and Radiology, Government College of Dentistry, Indore, Indore, IND; 2 Pediatric and Preventive Dentistry, Government Dental College and Hospital, Ahmedabad, IND; 3 Oral Medicine, Government College of Dentistry, Indore, Indore, IND

**Keywords:** subjective analysis, chemosensory dysfunction, palliative strategies, chemotherapy drugs, taste and smell changes

## Abstract

Objective: This study aimed to evaluate alterations in taste and smell perceptions among non-head and neck cancer patients receiving chemotherapy, aiming to identify factors influencing these changes.

Methods: A cohort of 70 non-head and neck cancer patients undergoing one to four cycles or more than four cycles, over a six-month period, from oncology outpatient clinics was recruited. Participants completed structured taste and smell questionnaires with assistance from interviewers. Demographic data, recurrence history, chemotherapy cycles, drug regimens, and taste and smell perceptions were analyzed using descriptive statistics and chi-square tests.

Results: The mean age of participants was 46.5 years, with a predominance of females (81.4%) and breast cancer cases (42.9%). Taste changes were more prevalent (62.9%) than smell changes (32.9%) post chemotherapy, particularly among those on combination drug regimens. Salty taste alterations were the most common (30.0%), followed by sweet taste (22.9%) and sour/bitter tastes (14.3%). Moreover, 38.57% of patients reported experiencing dysgeusia, while 30% noted the occurrence of parosmia post chemotherapy.

Conclusion: Chemotherapy-induced alterations in taste and smell significantly impact the quality of life and nutritional status of cancer patients. Despite often being overlooked, these changes warrant increased attention in oncological practice to inform treatment decisions and enhance symptom management, particularly in palliative care settings. Further research is needed to explore the implications of chemosensory alterations on patient outcomes and treatment strategies.

## Introduction

The chemical senses of taste and smell play a fundamental role in sustaining life. Disruptions in these senses are referred to as chemosensory dysfunction [1]. These alterations include decreased smell, such as anosmia (absence of odor perception), dysosmia (distorted ability to identify odors), parosmia (altered odor perception in the presence of another odor), hyposmia (decreased sensitivity to odor perception), phantosmia (odor perception without the presence of any odor), and agnosia (inability to discriminate perceived odors), and taste sensitivity (hypogeusia), complete loss of taste (ageusia), taste distortion (dysgeusia), or perceiving taste without actual stimuli (phantogeusia) [2]. These sensory changes often indicate symptoms of chronic diseases affecting multiple organ systems, including endocrine, metabolic, neurological, and others [3]. The exact mechanism of taste and smell abnormalities remains uncertain due to their multifactorial origins and the diverse cancer populations studied. Chemotherapy is often the primary treatment for cancer patients and has both immediate and long-term effects on these senses that are not entirely understood. Its non-selective nature affects not only cancer cells but also other rapidly dividing cells such as mucosal, olfactory, and gustatory receptors [4].

Chemosensory abnormalities significantly affect patients' nutritional status and quality of life during treatment [5]. Reduced appetite and sensitivity to taste and smell often result in inadequate nutrient intake, weight loss, and nutritional challenges [6], contributing to malnutrition, a prevalent issue among cancer patients that worsens with disease progression and increases morbidity and mortality in advanced stages [7].

In this study, we assessed changes in taste and smell perceptions among non-head and neck cancer patients undergoing chemotherapy. The findings from this research could contribute to the diverse palliative care strategies aimed at enhancing the overall quality of life for individuals with cancer.

## Materials and methods

Participants and procedure (data collection)

**Figure 1:**
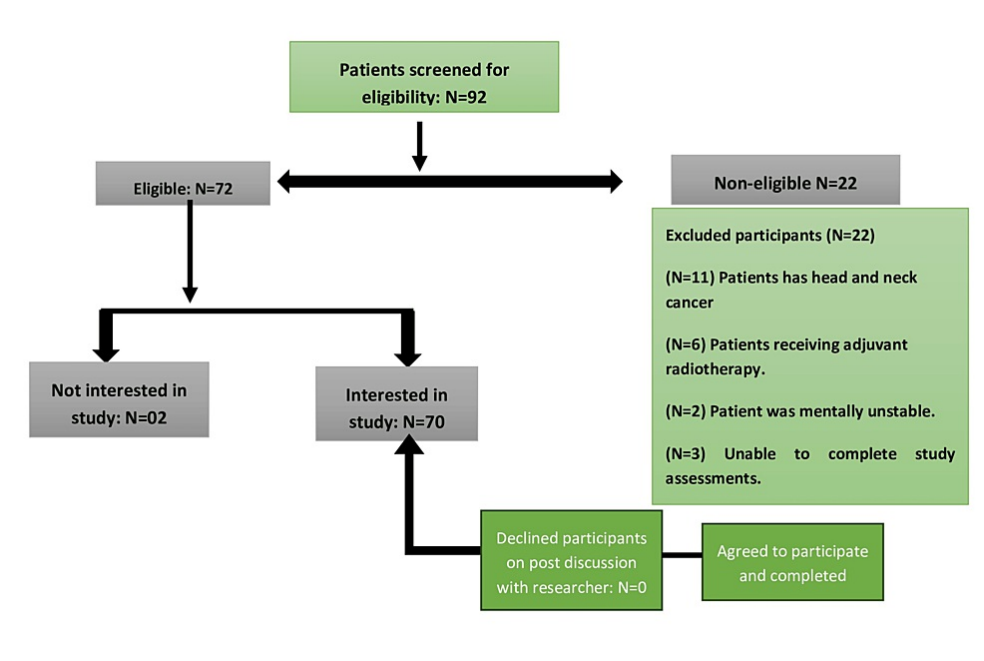
A summary of the recruitment and selection process

The main goal of this investigation is to recognize the factors that impact changes in taste and smell experienced by patients undergoing chemotherapy. The study employed an observational cross-sectional approach with a cohort of 70 consecutive non-head and neck cancer patients undergoing one to four cycles or more than four cycles, with a single or combination of drug regimens. Recruitment occurred over six months at oncology outpatient clinics in a tertiary care teaching hospital. The study's inclusion criteria were specific, targeting cancer patients aged 20 to 70 years undergoing chemotherapies at the oncology unit who provided witnessed signed consent forms. These criteria aimed to ensure the participation of a focused group for research purposes. Conversely, exclusion criteria were equally important in defining the study's boundaries. Patients with head and neck cancer, those undergoing radiotherapy or receiving adjuvant radiotherapy with chemotherapy, individuals diagnosed with dementia, those experiencing oral candidiasis, or those unable to complete study assessments were excluded. These criteria were designed to maintain the study's integrity by minimizing potential confounding factors and ensuring a more homogeneous participant group. Eligible individuals were approached after outpatient consultations or during chemotherapy planning sessions, where they received a brief information leaflet. The researcher then provided a detailed verbal explanation, addressed questions, and obtained written consent. Each participant underwent a 20-minute interview, completing structured taste and smell questionnaires (Table 1) with interviewer assistance. A summary of the recruitment and selection process is provided in Figure 1. The study protocol was approved by the Institutional Ethical Committee, Central Drugs Standard Control Organization, India (No.: 202/IEC/SS/2024).

**Table 1:** Interviewer-guided taste and smell questionnaire The questionnaire was formulated based on the self-reported experiences of patients post chemotherapy.

Characteristics	Options		
Demographic details (mark your answer by putting a cross mark ☒ in the box)			
1. Age:	☐ 20-30 years		
	☐ 30-40 years		
	☐ 40-50 years		
	☐ 50-60 years		
	☐ 60-70 years		
2. Sex:	☐ Male		
	☐ Female		
	☐ Others		
3. Cancer (history):	☐ 1^st^ time		
	☐ Recurrence		
4. Site of current cancer:	☐ Breast		
	☐ Ovary		
	☐ Lung		
	☐ Bladder		
	☐ Others		
5. Stage of cancer, based on clinical evaluation:	☐ Stage 1		
	☐ Stage 2		
	☐ Stage 3		
	☐ Stage 4		
6. Chemotherapy drug:	☐ Cisplatin		
	☐ Carboplatin		
	☐ Paclitaxel		
	☐ Combination of cisplatin and paclitaxel		
	☐ Combination of carboplatin and paclitaxel		
	☐ Other:		
7. Cycle of chemotherapy:	☐ Cycle 1		
	☐ Cycle 2		
	☐ Cycle 3		
	☐ Cycle 4		
	☐ More than 4		
Taste scale (mark your answer by putting a cross mark ☒ in the box)			
1. Is there any change in the sense of taste after chemotherapy?	☐ YES		
	☐ NO		
2. Is there any reduction in taste sensation post chemotherapy?	☐ Not at all		
	☐ Sometimes		
	☐ Always		
	☐ There is complete absence of taste		
Alteration in basic taste sensation (mark your answer by putting a cross mark ☒ in the box)			
Statement	Not at all	Sometimes	Always
1. Is there any alteration in bitter taste post chemotherapy?	☐	☐	☐
2. Is there any alteration in sweet taste post chemotherapy?	☐	☐	☐
3. Is there any alteration in sour taste post chemotherapy?	☐	☐	☐
4. Is there any alteration in salty taste post chemotherapy?	☐	☐	☐
5. Is there any sense of taste even if there is no food in the mouth?	☐	☐	☐
6. Is there a persistent bad taste in the mouth?	☐	☐	☐
Smell scale (mark your answer by putting a cross mark ☒ in the box)			
1. Is there any change in the sense of smell after chemotherapy?	☐ YES		
	☐ NO		
2. Is there any reduction in odor perception post chemotherapy?	☐ Not at all		
	☐ Sometimes		
	☐ Always		
	☐ There is complete absence		
Alteration in smell sensation (mark your answer by putting a cross mark ☒ in the box)			
Statement	Not at all	Sometimes	Always
3. Is there any change in normal perception of odor (triggered by a stimulus)?	☐	☐	☐
4. Is there any perception of smells that are not really there (occurs without a stimulus)?	☐	☐	☐

Subjective assessment

Patients self-assessed taste and smell changes through a questionnaire, evaluating alterations in salty, sweet, sour, and bitter tastes, including their intensity and nature (e.g., stronger, weaker, and taste hallucination). Taste alteration was defined as a positive response to taste changes in the questionnaire at each assessment. Smell alterations were recorded as present or absent. Subjective taste alterations for each taste type were measured using a Likert scale (always, sometimes, or never) featuring closed-ended questions.

The interviewer-guided survey encompasses demographic information of patients, recurrence history, the number of completed chemotherapy cycles, details of the drug regimen, a taste survey covering sweetness, bitterness, sourness, and spiciness, as well as assessments for phantogeusia and smell. All gathered data were compiled, analyzed, and tabulated using SPSS software (IBM Corp., Armonk, NY). Notably, the questionnaire has been formulated in the Hindi language to ensure comprehension by local patients. Participants were asked to provide scores for changes observed after undergoing one to four cycles or more than four cycles, with specific reference to days following each cycle. Responses to these questions were analyzed inductively by content analysis.

Data analysis

Data analysis was done using SPSS version 21.0. Descriptive statistics were performed. Inter-group comparison of categorical variables was done using the chi-square test and continuous variables were done using independent t-test/Mann-Whitney U test and/or one-way ANOVA/Kruskal-Wallis test. P-value < 0.05 was considered statistically significant.

## Results

**Figure 2:**
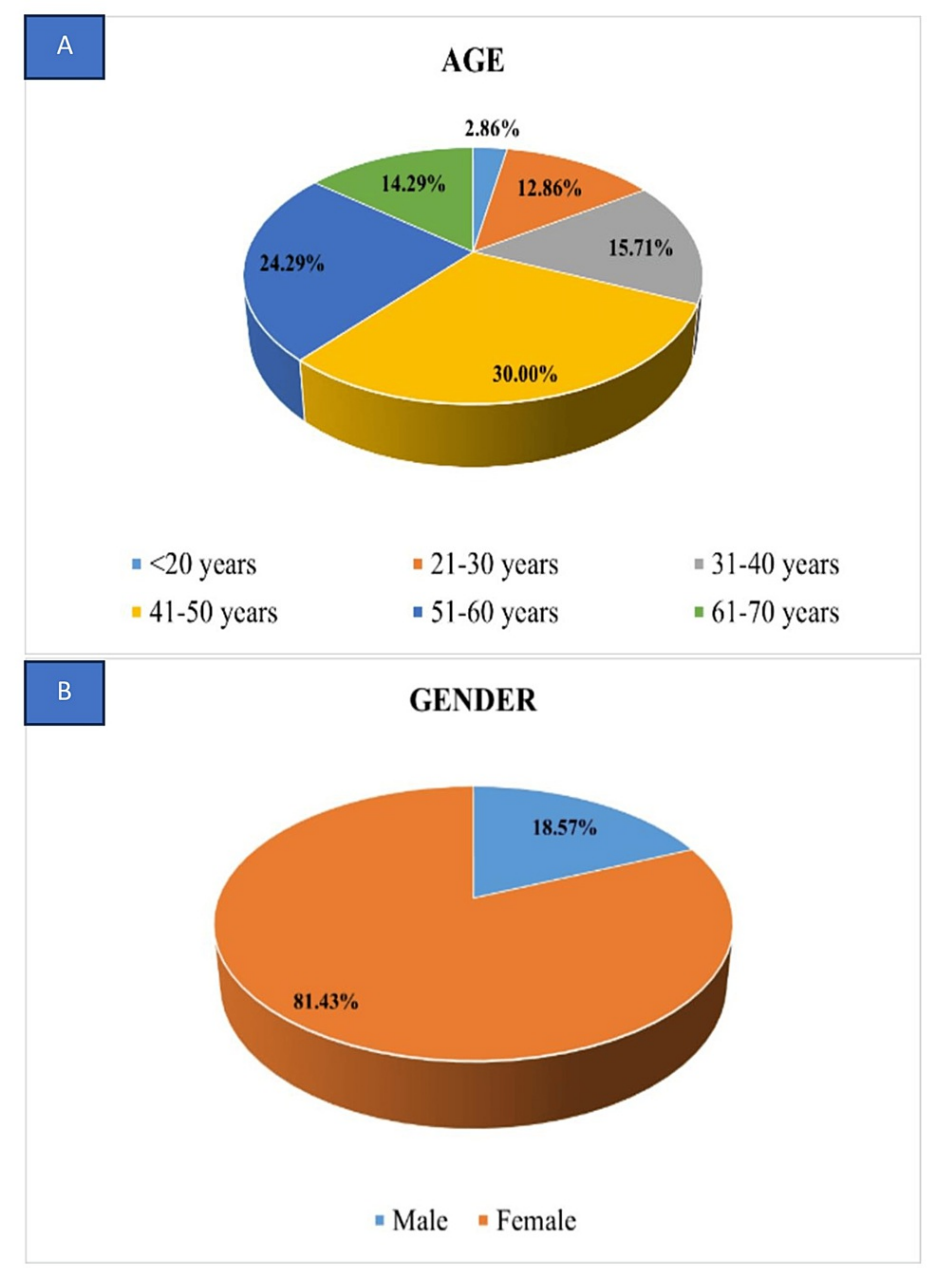
Distribution of participants based on (A) age and (B) gender

**Figure 3:**
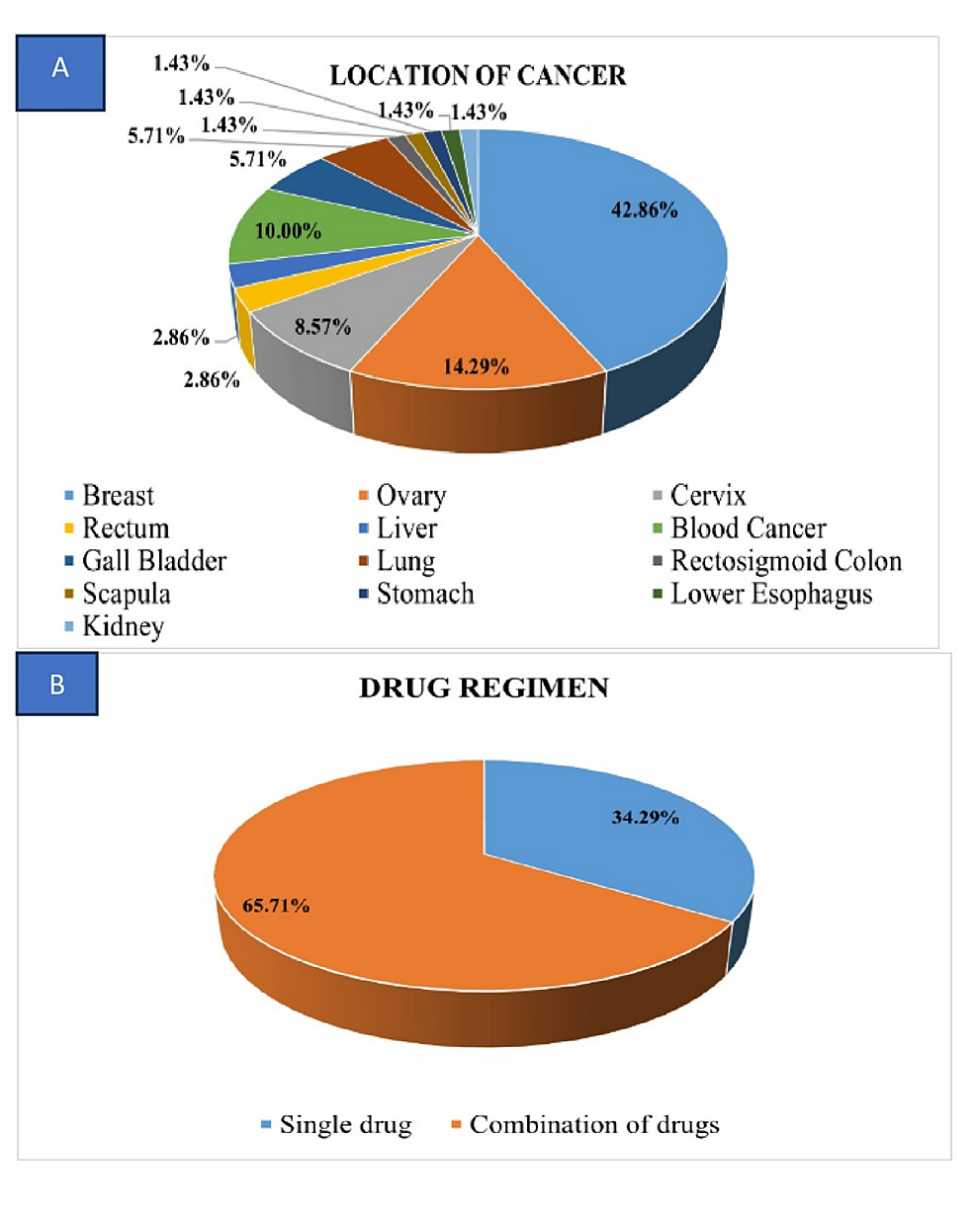
Distribution of participants based on (A) location of cancer and (B) drug regimen

**Figure 4:**
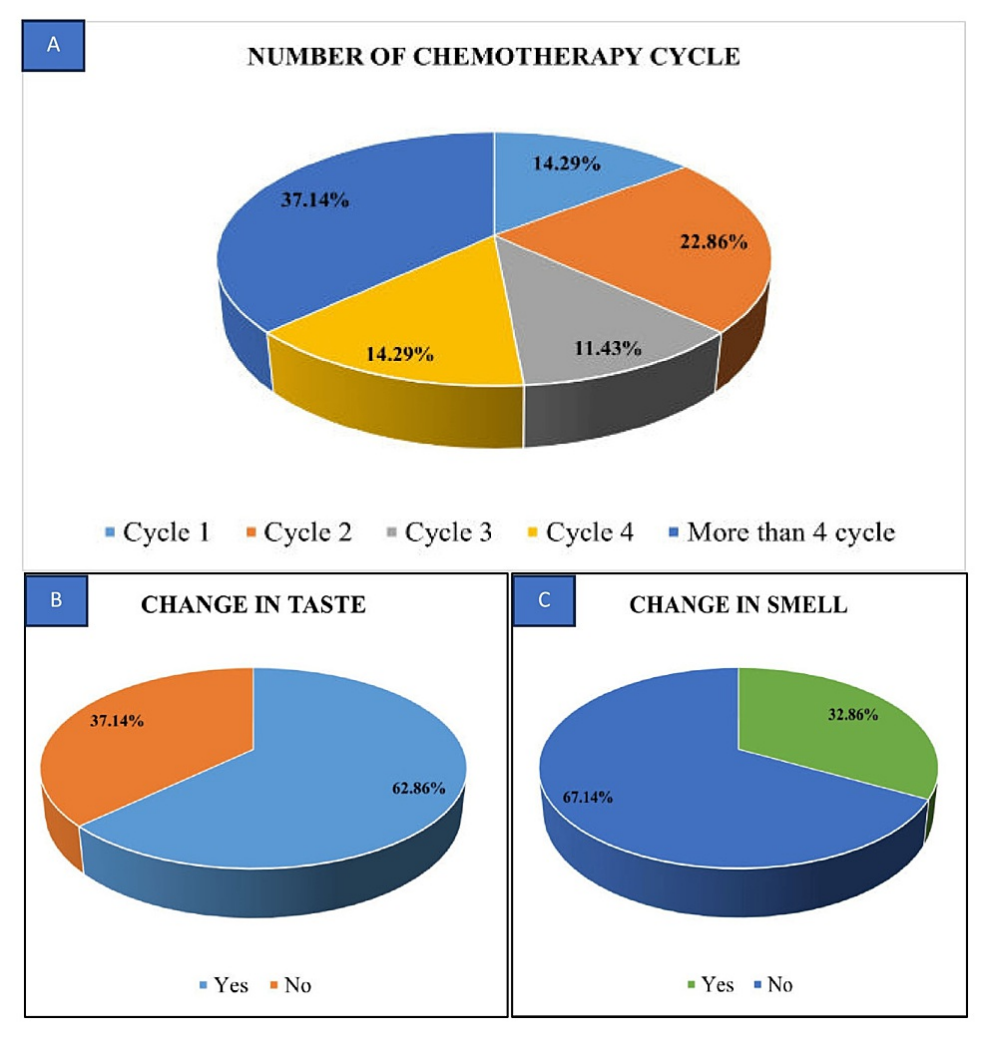
Distribution of participants based on (A) number of chemotherapy cycles, (B) change in taste, and (C) change in smell

The research comprised a cohort of 70 patients undergoing chemotherapy, with a mean age of 46.5 ± 13.174 years. The average duration for the onset of taste and smell alterations was recorded as 4.6 ± 2.66 days. Of the participants, 57 were females, constituting 81.4%, while 13 were males, accounting for 18.6% (Figure 2). Breast cancer was the predominant malignancy site at 42.9%, followed by ovarian cancer at 14.3%. The majority of patients (92.9%) were experiencing cancer for the first time, with only 7.1% having a recurrence. Regarding the drug regimen, 65.7% (46) were under a combination of drugs, while 34.3% (24) were on a single drug (Figure 3). Among the subjects, 37.1% had undergone more than four cycles of chemotherapy, followed by cycle 2 than cycle 3 (Table 2). It has also been observed that individuals who came within one to three days after any cycle of chemotherapy experienced more pronounced taste and smell changes compared to those who visited after four days (Figure 4A).

**Table 2:** Data depicting the demographic details included in the study

Age group	Number of subjects	Percentage
<20 years	2	2.9
21-30 years	9	12.9
31-40 years	11	15.7
41-50 years	21	30.0
51-60 years	17	24.3
61-70 years	10	14.3
Days post chemotherapy when the maximum change in taste & smell was experienced	4.6 ± 2.66 days	_
Gender		
Male	13	18.6
Female	57	81.4
Location		
Breast	30	42.9
Ovary	10	14.3
Cervix	6	8.6
Rectum	2	2.9
Liver	2	2.9
Blood cancer	7	10.0
Gall bladder	4	5.7
Lung	4	5.7
Rectosigmoid colon	1	1.4
Scapula	1	1.4
Stomach	1	1.4
Lower esophagus	1	1.4
Kidney	1	1.4
History of cancer		
1^st^ time	65	92.9
Recurrence	5	7.1
Drug regimen		
Single drug	24	34.3
Combination of drugs	46	65.7
Drug regimen		
Cycle 1	10	14.3
Cycle 2	16	22.9
Cycle 3	8	11.4
Cycle 4	10	14.3
More than 4 cycles	26	37.1

In the analysis of chemotherapeutic drug regimens, the data revealed that the predominant occurrence among patients was taste changes (62.9%), while a lesser proportion displayed alterations in smell (32.9%) post chemotherapy (Figures 4B, 4C). Conspicuously, taste changes were more prevalent in individuals receiving combination drug regimens (65.2%) compared to those administered single drug regimens (58.3%) (Figure 3B). Similarly, instances of smell alterations were predominantly observed in subjects undergoing combination drug regimens (33.3%) relative to those subjected to single drug regimens (32.6%) (Table 3). Taste changes were notably more prevalent among patients undergoing combination drug regimens (65.2%), particularly among 11 individuals receiving a combination of carboplatin and paclitaxel, followed by docetaxel and cyclophosphamide, compared to those on single drug regimens (58.3%), with nine patients receiving paclitaxel, followed by docetaxel alone. Furthermore, smell alterations were predominantly observed in patients undergoing combination drug regimens (33.3%) compared to those on single drug regimens (32.6%), with 15 cases and eight cases, respectively (Table 4). These continuous variables were analyzed by using an independent t-test/Mann-Whitney U test and/or one-way ANOVA/Kruskal-Wallis test.

**Table 3:** Data depicting the changes in taste and smell sensations among the participants

Taste alteration		Scale		Frequency (n)	Percentage (%)	
Change in basic taste						
Sweet		Always		14		20.0
		Sometimes		2		2.9
		No		54		77.1
Sour		Always		10		14.3
		Sometimes		0		0.0
		No		60		85.7
Bitter		Always		10		14.3
		Sometimes		0		0.0
		No		60		85.7
Salty		Always		17		24.3
		Sometimes		4		5.7
Dysgeusia		No		49		70.0
		Always		27		38.6
		Sometimes		5		7.1
Phantogeusia			Yes	2		2.9
			No	68		97.1
Change in smell						
Yes				23		32.9
No				47		67.1
Phantosmia	Always			0		0.0
	Sometimes			0		0.0
	No			70		100.0

**Table 4:** Data depicting the correlation between the chemotherapeutic drug regimen and taste & smell changes

Prevalent chemotherapeutic drug regimens in the study	No. of patients receiving drug regimen (n)	No. of patients who experienced taste changes (n)	No. of patients who experienced smell changes (n)
Combination drug regimen			
Carboplatin + paclitaxel	11	7	4
Docetaxel + cyclophosphamide	4	4	1
Cyclophosphamide + epirubicin + fluorouracil	3	2	2
Single drug regimen			
Paclitaxel	9	7	3
Docetaxel	5	2	0
Letrozole	2	1	1
Total	34	23	11

Concerning taste alterations, 36.4% (16 out of 44) reported consistent hypogeusia, 6.8% (three out of 44) occasionally experienced hypogeusia, and 4.5% (two out of 44) consistently faced ageusia post chemotherapy. Salty taste alteration (30.0%) was the most common, followed by sweet taste (22.9%) and sour and bitter taste (14.3%) in the analysis of taste sensations (Figure 4).

**Figure 5:**
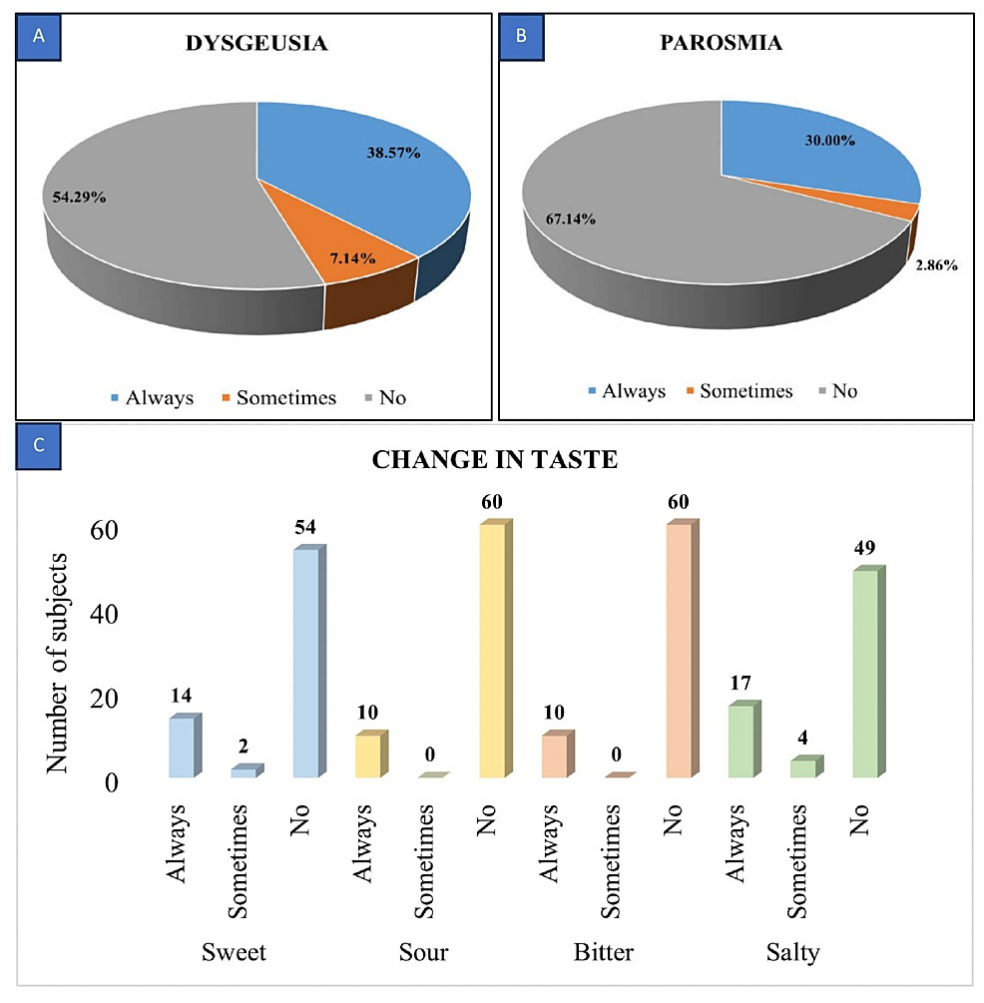
Distribution of study subjects based on the number of the presence of (A) dysgeusia, (B) parosmia, and (C) change in taste

Dysgeusia was consistently present in 38.6% of subjects due to chemotherapy, while 54.3% showed no signs of dysgeusia. Phantogeusia was observed in only two older subjects, suggesting a potential lack of association with the effects of chemotherapy. Among subjects experiencing changes in smell sensation, the majority (67.1%) reported no impact from chemotherapy. However, most of the cancer patients (47, 67.1%) did not have parosmia, followed by 21 (30%) patients who always had parosmia, and only two (2.9%) sometimes had parosmia (Figure 5). In the comparison of taste and smell changes between subjects receiving a single drug and those on combination drug regimens, the latter exhibited more alterations. The p-value, however, was >0.05, indicating no significant association between drug regimen and changes in taste or smell sensations. This inter-group comparison of categorical variables will be done using the chi-square test. The results have been tabulated to show a comprehensive overview of the study, encompassing various variables along with their corresponding statistical analyses in Tables 2-4.

## Discussion

Cancer treatment contributes to malnutrition in over 40% of hospitalized patients, linked to unfavorable outcomes, reduced therapy response, and diminished quality of life. A study found that 45% of hospitalized adults [4] lost 10% of their body weight, with 25% experiencing a loss of 20% or more [4], highlighting the impact of taste disorders on malnutrition in cancer patients [8]. Taste and smell disorders in cancer patients are often overlooked, possibly due to the infrequent use of standardized taste tests by healthcare providers. Additionally, many taste disorders do not fit conventional taste categories [9]. The literature recognizes five basic tastes: sweet, sour, bitter, salty, and umami [10]. Taste begins with the activation of receptors on microvilli, forming taste buds. These modified epithelial cells can process gustatory, olfactory, and trigeminal stimulation [11]. Smell disorders are classified into anosmia (absence of smell perception), hyposmia (quantitatively reduced scent perception), parosmia (qualitative distortion of a detected smell), and phantosmia (perception of odors in the absence of stimuli) [12,13]. This survey involved 70 cancer patients undergoing various treatments to investigate if chemotherapeutic drugs alter chemosensory perception. The questionnaire-based subjective analysis traversed six months for cancer patients (excluding head and neck) undergoing chemotherapy with one to four or more cycles, and patients reported their experiences on different days after each cycle.

In this investigation, the predominant age group among patients undergoing chemotherapy was 41-50 years (30%). This demographic pattern mirrors findings from the 2009 study by Bernhardson et al. [14]. Additionally, McGettigan et al. conducted a study in 2019 involving 510 and 30 patients, respectively [15]. Their research revealed the highest prevalence of affected individuals in the age groups of 55-64 and >65 years, respectively [15]. In terms of gender, female (81.4%) patients were the predominant group, consistent with findings by McGettigan et al. (2019) [15] and Kano et al. (2013) [16].

Categorized by the specific cancer site, the preponderant majority of subjects exhibited instances of breast cancer (42.9%), succeeded by ovarian cancer (14.3%). These observations align with the findings reported in studies by Steinbach et al. (2009) [17], Sozeri et al. (2015) [18], and Bernhardson et al. (2008) [14]. Conversely, Zabernigg et al. (2010) [19] and Belqaid et al. (2014) [20] identified the lung as the predominant site for malignancy. Most of the subjects had cancer for the first time (65, 92.9%), and only five (7.1%) had a recurrence of cancer. Similarly, Steinbach et al. did a study on 87 cancer patients, of whom only eight (9%) had recurrence [17].

Analysis of the chemotherapeutic drug regimen revealed that the majority of patients experienced taste changes (62.9%), with only 23 (32.9%) exhibiting smell changes post chemotherapy. A parallel study conducted by Amézaga et al. in 2018 reported comparable results, with 76% of patients reporting taste disorders and 45% reporting changes in smell [4].

**Figure 6:**
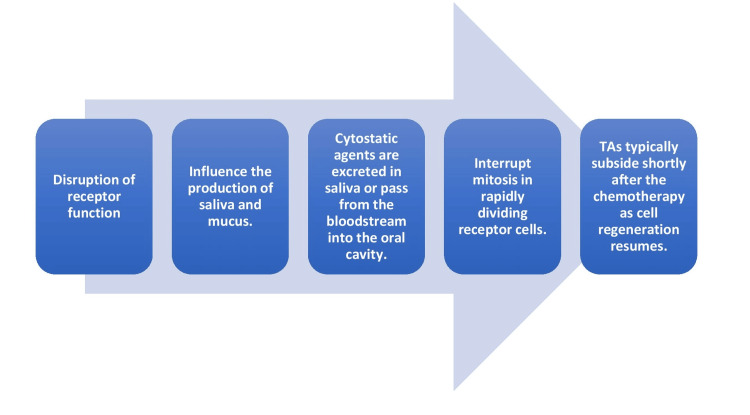
Mechanism of action of chemotherapy-induced taste alteration Figure created by the authors.

Taste and smell changes were significantly more common among patients undergoing combination drug regimens, particularly those including paclitaxel, carboplatin, or cisplatin, compared to those on single drug regimens. Similarly, Sozeri et al. in 2015 found that combination chemotherapeutic regimens, notably including paclitaxel, Herceptin, gemcitabine, and cisplatin, were associated with the highest incidence of taste alterations in patients [18]. The exact processes through which cytostatic agents induce taste alterations (TAs) remain somewhat unclear. One possible mechanism is explained below in Figure 6 [19].

Amongst the subjects included in the study, the majority had more than four cycles of chemotherapy (37.1%) as reported by Denda et al. [21].

Based on the number of the presence of change in taste subjects with a general change in taste sensation, 36.4% reported that they always experienced hypogeusia, 6.8% sometimes experienced hypogeusia, and only 4.5% always experienced ageusia after chemotherapy. Similar findings were seen in a study done by Kano et al. in 2013 [16]. In contrast to their finding, our study showed only 2.9% of subjects had phantogeusia.

In our study, the most prevalent taste alteration post chemotherapy was found to be salty taste (30.0%), followed by sweet taste (22.9%), and then sour and bitter taste (14.3%). Similar results were found in the research done by Turcott et al. in 2016 [22] and Steinbach et al. in 2009 [17]. However, our findings differ from those of Rehwaldt et al. (2009) [23], who indicated that the most commonly affected taste was bitter, followed by sour taste. Additionally, a significant number of patients in their study reported experiencing a metallic taste after chemotherapy.

**Figure 7:**
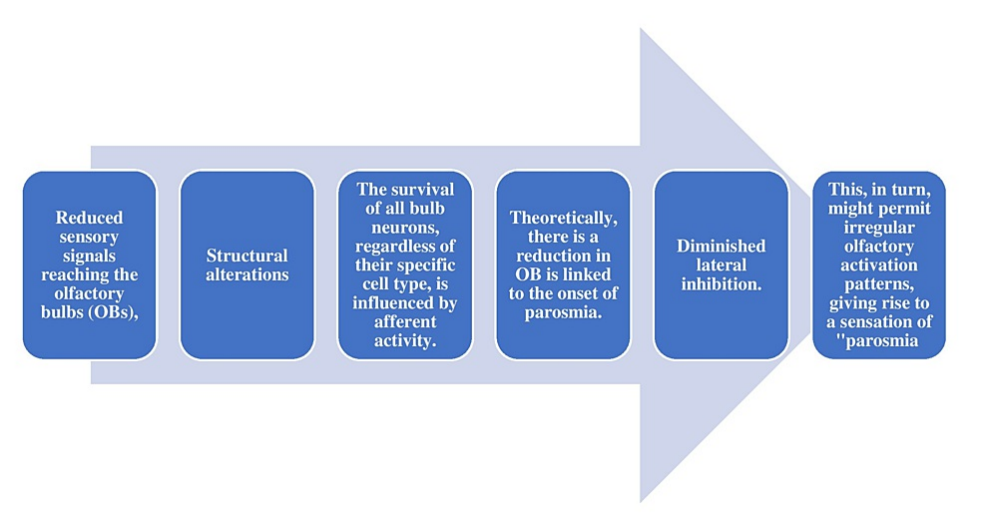
The mechanism behind the pathogenesis of parosmia after chemotherapy Figure created by the authors.

As already described, taste alterations have more prevalence than smell changes post chemotherapy in our study; hence, only 32.9% show smell changes. However, the majority of cancer patients after chemotherapy do not exhibit parosmia, with only 30% consistently experiencing it, predominantly observed in elderly patients. This aligns with the findings of Haxel et al. (2016) [24], who conducted a study on 28 patients using the Sniffin' Sticks test. The mechanism behind the pathogenesis of parosmia after chemotherapy is explained in the chart below(Figure 7) [25].

A limitation of the study is the lack of specificity regarding the drug regimens contributing to chemosensory alterations following chemotherapy. Moreover, the study does not include comparisons of chemosensory changes before, during, and after chemotherapy cycles.

## Conclusions

Taste and smell alterations in chemotherapy patients are often overlooked, warranting increased attention in daily oncological practice and research. Providing patients with information about this side effect is crucial and should be a key factor in clinical decision-making. This is especially significant in palliative care settings, where the primary therapeutic goal is symptom control, and the impact of treatment on quality of life is pivotal in deciding on specific chemotherapy regimens. Striking the right balance between treatment benefits and burdens is crucial to avoid unnecessary strain on patients. Further research is essential to thoroughly investigate taste and smell alterations in cancer patients, their impact on quality of life, and their implications for treatment strategies.
